# Modulation of calcium-induced cell death in human neural stem cells by the novel peptidylarginine deiminase–AIF pathway

**DOI:** 10.1016/j.bbamcr.2014.02.018

**Published:** 2014-06

**Authors:** Kin Pong U, Venkataraman Subramanian, Antony P. Nicholas, Paul R. Thompson, Patrizia Ferretti

**Affiliations:** a Developmental Biology Unit, UCL Institute of Child Health, London WC1N 1EH, UK; b Department of Chemistry, TSRI, Scripps Florida, FL 33458, USA; c Department of Neurology, University of Alabama at Birmingham and Birmingham VA Medical Center, Birmingham, AL 35294, USA

**Keywords:** Apoptosis inducing factor (AIF), Cell death, Citrullination–deimination, Human neural stem cell, Peptidylarginine deiminase (PAD, PADI), Vimentin

## Abstract

PADs (peptidylarginine deiminases) are calcium-dependent enzymes that change protein-bound arginine to citrulline (citrullination/deimination) affecting protein conformation and function. PAD up-regulation following chick spinal cord injury has been linked to extensive tissue damage and loss of regenerative capability. Having found that human neural stem cells (hNSCs) expressed PAD2 and PAD3, we studied PAD function in these cells and investigated PAD3 as a potential target for neuroprotection by mimicking calcium-induced secondary injury responses. We show that PAD3, rather than PAD2 is a modulator of cell growth/death and that PAD activity is not associated with caspase-3-dependent cell death, but is required for AIF (apoptosis inducing factor)-mediated apoptosis. PAD inhibition prevents association of PAD3 with AIF and AIF cleavage required for its translocation to the nucleus. Finally, PAD inhibition also hinders calcium-induced cytoskeleton disassembly and association of PAD3 with vimentin, that we show to be associated also with AIF; together this suggests that PAD-dependent cytoskeleton disassembly may play a role in AIF translocation to the nucleus. This is the first study highlighting a role of PAD activity in balancing hNSC survival/death, identifying PAD3 as an important upstream regulator of calcium-induced apoptosis, which could be targeted to reduce neural loss, and shedding light on the mechanisms involved.

## Highlights

• PAD pathway is a novel regulator of cell death/survival in human neural stem cells.
• PAD3 inhibition reduces calcium-induced cell death and cytoskeleton disassembly.
• PAD3 is required for AIF (apoptosis inducing factor) translocation to the nucleus.
• Targeting the PAD pathway could provide a novel strategy for neuroprotection.

## Graphical abstract

**Figure:**
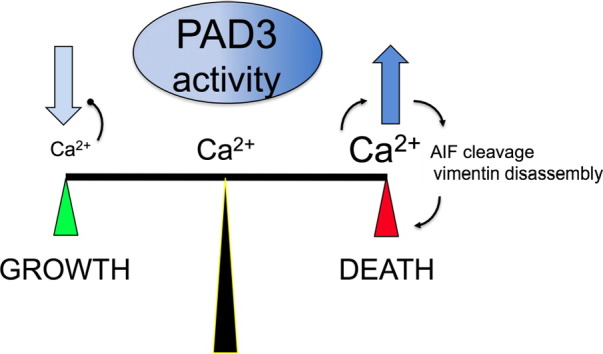

## 1 Introduction

The importance of citrullination/deimination, the hydrolysis of protein-bound arginine to citrulline, in several neural pathologies is becoming increasingly apparent [1–7]. These include traumatic injury, hypoxia and neurodegenerative diseases, such as Alzheimer's disease and multiple sclerosis, where an increase in Ca^2 +^ levels is considered to play a relevant role in neural tissue loss [8–11].

Citrullination is carried out by a family of calcium-dependent enzymes, peptidylarginine deiminases (PADs) that have different tissue distributions, often overlapping, and are believed to have distinct substrate specificity [12–17]. PAD activity has been reported in the cytoplasm, including mitochondrial and microsomal fractions, as well as in the nucleus [18]. Among the known PAD substrates are cytoskeletal proteins and histones [19,20]. PAD2, the ancestral and more widely expressed PAD, is the main PAD in the central nervous system (CNS), though expression of other PADs has been reported in various neural cell types [15,21,22].

Inhibition of citrullination can reduce disease onset or severity in mouse models of multiple sclerosis, rheumatoid arthritis and ulcerative colitis [23–25]. Significantly, the PAD inhibitor Cl-amidine reduces neural damage in a chick spinal cord injury model and in a neonatal mouse hypoxia model [6,7,26]. In the chick, PAD3 appears to be the main PAD involved in secondary injury response, that includes increased intracellular Ca^2 +^, leading to apoptosis and consequent neural tissue loss. Disruption of a known PAD target, vimentin, reduces viability of HEL cells, an effect inhibited by Ca^2 +^ chelators [27,28]. Ca^2 +^-dependent cell death is not executed by caspase 3, and translocation to the nucleus of the mitochondrial protein, apoptosis inducing factor (AIF), mediates apoptosis in injured brains [29,30]. AIF down-regulation appears to be neuroprotective; hence effective targeting of this pathway could be valuable in pathologies involving Ca^2 +^ homeostasis dysregulation either during embryonic development or post-natally, such as traumatic injuries, hypoxia–ischemia and damage to neural precursors caused by radiotherapy in young brains [7,30–35].

Having first established that PAD3 is expressed in the developing human nervous system and in human neural stem cells, as in the chick [6], we investigated whether this pathway may be a novel key regulator of human neural cell death/survival focusing on its potential involvement in calcium-induced cell damage. We show that whereas PAD inhibition significantly increases hNSC growth, raising intracellular Ca^2 +^ with thapsigargin increases PAD3 activity and cell death that is greatly reduced by a PAD inhibitor or PAD3 siRNA. We also show that thapsigargin-induced death in hNSCs is dependent on AIF, not caspase 3, and that cleavage of AIF, required for its translocation to the nucleus, is PAD3-dependent. Finally, we show that vimentin becomes associated with PAD3 upon cytoplasmic Ca^2 +^ increase, that disrupts cytoskeleton integrity, and that vimentin is also associated with AIF, suggesting a possible role in AIF stabilization in the mitochondria. Altogether our findings support a role for human PADs in the regulation of cell death/survival, identify PAD3 as an upstream regulator of Ca^2 +^-dependent cell death, and shed light on the pathway(s) involved.

## 2 Materials and methods

### 2.1 Cell lines and treatments

Human tissues were supplied by Human Developmental Biology Resources under ethical approval. Human neural stem cell lines (hNSCs) from either the brain or the spinal cord of embryos between Carnegie stage (CS) 18 (gestation age 37–42 days) and CS22 (gestational age 54–56 days) were established and grown as previously described [36]. HEK293T (Human Embryonic Kidney 293) cells were grown DMEM–High Glucose–GlutaMAX (Gibco) supplemented with 10% fetal calf serum. The PAD inhibitor Cl-amidine [37] was dissolved in phosphate buffer saline (PBS) and used at different concentrations (0.1–500 μM final concentration). Thapsigargin (Sigma) dissolved in ethanol was used at different concentrations (1–25 μM final concentration). Cells were treated with Cl-amidine 15 min before the addition of thapsigargin. In some experiments the inhibitory effect of Cl-amidine treatment 15 min after the addition of thapsigargin was also assessed.

### 2.2 In-situ hybridization

Spinal cords from human embryos at 46 days of gestation were fixed in 4% PFA (par-formaldehyde), paraffin embedded and sectioned (7 μm thickness). PAD2 and PAD3 probes were produced by PCR amplification of the regions corresponding to exon 1 and exon 7 of PAD2 and PAD3 cDNAs, and ligation into the pGEMT-Easy vector (Promega). The pGEMT-Easy vectors containing either the PAD2 or PAD3 insert were digested with SpeI or NcoI restriction endonuclease and transcribed using T7 and Sp6 RNA polymerase to produce DIG labeled riboprobes.

In situ hybridization was carried out using digoxigenin-labeled riboprobes essentially as previously described [38]. In brief, de-waxed sections digested with proteinase K (20 mg/ml) were re-fixed in 4% PFA solution, treated with 0.1 M triethanolamine containing 0.25% acetic anhydride and hybridized overnight at 65°°C. After high stringency washes, the riboprobes were localized using an alkaline phosphatase-conjugated sheep anti-digoxigenin Fab fragment (Roche) and detected by incubation in nitroblue tetrazolium/5-bromo-4-chloro-3-indolyl phosphate (NBT/BCIP, Roche).

### 2.3 Immunocytochemistry

hNSCs plated on either coverslip or chamber-slides (PAA) were fixed with either 4% PFA or 100% ice cold methanol depending on the antigen to be detected, for 15 and 5 min, respectively. Immunocytochemistry was carried out at room temperature essentially as previously described [39]. The primary antibodies used were rabbit anti-PAD3 (Covalab), rabbit anti-PAD2 (Covalab), rabbit anti-Cleaved Caspase 3 (Cell Signaling), goat anti-AIF (Santa Cruz), mouse anti-ß3-tubulin (Promega), mouse anti-vimentin (Dako), and Alexa 568-conjugated anti-Annexin V (Life Technologies). Filamentous actin was detected with Alexa Fluor 488-conjugated phalloidin (0.02 unit/μl; Invitrogen). The secondary antibodies were donkey anti-mouse Alexa 488, donkey or goat anti-rabbit Alexa 488 or 568, and donkey anti-goat Alexa 594 (Life Technology). Nuclei were counterstained with Hoechst 33258 (2.5 μg/ml final concentration). Stained cells were visualized and digitally scanned using an Axiophot 2 (Zeiss) with Hamamatsu ORCA-ER digital camera or by confocal laser scanning microscopy using an LSM 710 (Zeiss). Image collection and analysis were performed using Openlab (Perkin Elmer-Improvision) or ImageJ software [40].

### 2.4 Western blot and immunoprecipitation

Proteins were extracted from either cell pellets or tissues as previously described [41]. For Western-blot analysis, 20–40 μg of proteins per lane were separated by 10% or 12% SDS-PAGE and electrotransferred to nitrocellulose membranes (GE Healthcare) using a TransBlot-SD (BioRad). The primary antibodies used were the same as for immunocytochemistry. The secondary antibodies were: horseradish peroxidase-conjugated anti-rabbit immunoglobulin IgG (Dako, Denmark; 1:4000) and anti-mouse immunoglobulin IgM (Serotec, 1: 4000). Bound antibodies were visualized using the ECL Western Blotting reagents (Amersham Biosciences).

Immunoprecipitation was carried out using the Millipore 17-500 Catch and Release Immunoprecipitation kit according to the manufacturer's instructions (Upstate). Three hundred to seven hundred microgram of hNSC proteins were immunoprecipitated with the anti-PAD3 antibody (5 μg), the anti-AIF antibody (2 μg), or the F95 antibody (10 μg) and the immunoprecipitated fractions analyzed by Western blotting.

### 2.5 Assessment of cell growth cell growth and death

The methylene blue (MB) assay was used to carry out cell growth analysis in 96 well plates as previously described [42]. Propidium iodide (PI, 2 μg/ml final concentration; Sigma) staining was used to assess cell death either in live cultures or in PFA-fixed cells. Nuclei were counterstained with Hoechst 33258. Staining was visualized either using a fluorescent inverted microscope (Olympus 1X71) with a monochrome ORCA R^2^ digital camera (Hamamatsu) or as for immunocytochemistry.

### 2.6 PAD activity assay

The BAEE (Nα-benzoyl-l-arginine ethyl ester; Sigma) colorimetric assay was used to assess PAD activity in 5 μg (1 μg protein/μl) of protein extract per sample with minor modifications from previously described protocols [43]. The optical density of the colorimetric reaction detecting citrulline was measured at 490 nm using a microplate reader (Revelation v4.21 Dynex Technologies, Inc). The background (reading at time point 0) was subtracted from each sample measurement.

### 2.7 Reverse-transcription-polymerase chain reaction (RT-PCR) and quantitative real-time polymerase chain reaction (qPCR)

Total RNA was extracted from tissues (for developmental studies at least three human embryos at each stage of gestation were used) and cells using TRIzol reagent (Invitrogen), according to manufacturer's instruction, and MLTV-Reverse Transcriptase (Promega) were used to prepare cDNA [44]. In reverse transcription-polymerase chain reaction experiments, all cDNAs were amplified for 35 cycles except for the house-keeping gene GAPDH (glyceraldehyde-3-phosphate dehydrogenase; 30 cycles) using the following conditions: 5 min 95 °C, 30 s 95 °C, 30 s 52 °C–68 °C (depending on primers), and 5 min 72 °C and 4 °C. PCR products were resolved on 1.5% agarose gel. Primer sequences for RT-PCR and qPCR and amplification conditions used are shown in Supplementary Table 1.

Total RNA was extracted from tissues (for developmental studies at least three human embryos at each stage of gestation were used) and cells using TRIzol reagent (Invitrogen), according to manufacturer's instruction; MLTV-Reverse Transcriptase (Promega) was used to prepare cDNA [44]. In reverse transcription-polymerase chain reaction experiments, all cDNAs were amplified for 35 cycles except for the house-keeping gene GAPDH (glyceraldehyde-3-phosphate dehydrogenase; 30 cycles) using the following conditions: 5 min 95 °C, 30 s 95 °C, 30 s 52 °C–68 °C (depending on primers), and 5 min 72 °C and 4 °C. PCR products were resolved on 1.5% agarose gel. Primer sequences for RT-PCR and qPCR and amplification conditions used are shown in Supplementary Table 1.

### 2.8 PAD3 overexpression and siRNA knockdown

The pET16b-hPAD3 plasmid [45] was used as a template for PCR amplification of human PAD3 (PADI3) using forward and reverse primers that contained BglII and EcoRI restriction sites, respectively, and ligated into EGFP-N2 plasmid using these restriction sites. The PAD3 construct lacking the enzyme active site, ΔPAD3-EGFP, was made by digesting the PAD3-EGFP plasmid sequentially with Eco53kI restriction endonuclease at 37 °C and SmaI restriction endonuclease at 24 °C. Constructs were sequenced to ensure they contained the correct sequences.

HEK293T and hNSC at 60% confluency were transfected with the PAD3-EGFP, ΔPADI3-EGFP or EGFP only plasmid using Lipofectamine LTX (Invitrogen). For siRNA inhibition studies, hNSC cells were transfected with validated human PADI3 siRNA (s28546), or PADI2 siRNA (s223214), or negative control siRNA 2 (Ambion) at a final concentration of 250 nM with Lipofectamine LTX. Knockdown efficiency was assessed by RT-qPCR analysis.

### 2.9 Statistical analysis

Each experiment was performed at least 3 times; each experimental group was n = 3–6. Statistical significance was evaluated by ANOVA and Student's *t*-test; p < 0.05 was taken to be significant.

## 3 Results

### 3.1 PAD2 and PAD3 isozymes are expressed in the developing human nervous system and in human neural stem cells (hNSCs)

**Fig. 1:**
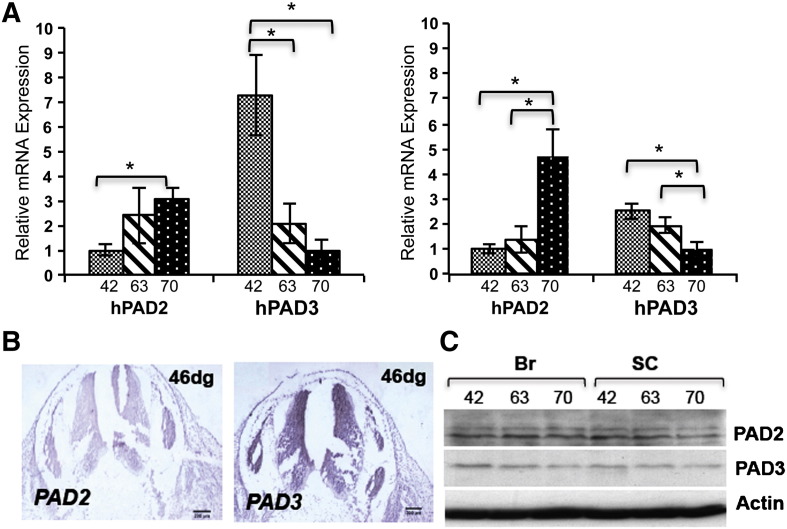
PAD expression in the developing human central nervous system (brain and spinal cord) and in hNSCs. A) Real time RT-PCR analysis of PAD3 and PAD2 in fetal brains (left panel) and spinal cords (right panel) from human embryos at 42, 63 and 70 days of gestation. PAD2 expression increases while PAD3 expression decreases with development. Human liver was used as a positive control for all PADs. Asterisk indicates statistically significant differences (p < 0.05). B) PAD2 and PAD3 transcript detected by in situ hybridization in human spinal cord at 46 days of gestation (dg). Scale bars are 200 μm. C) PAD2 and PAD3 protein detected by Western blot in developing human brain (Br) and spinal cord (SC); no dramatic change in PAD protein expression is observed.

**Fig. 2:**
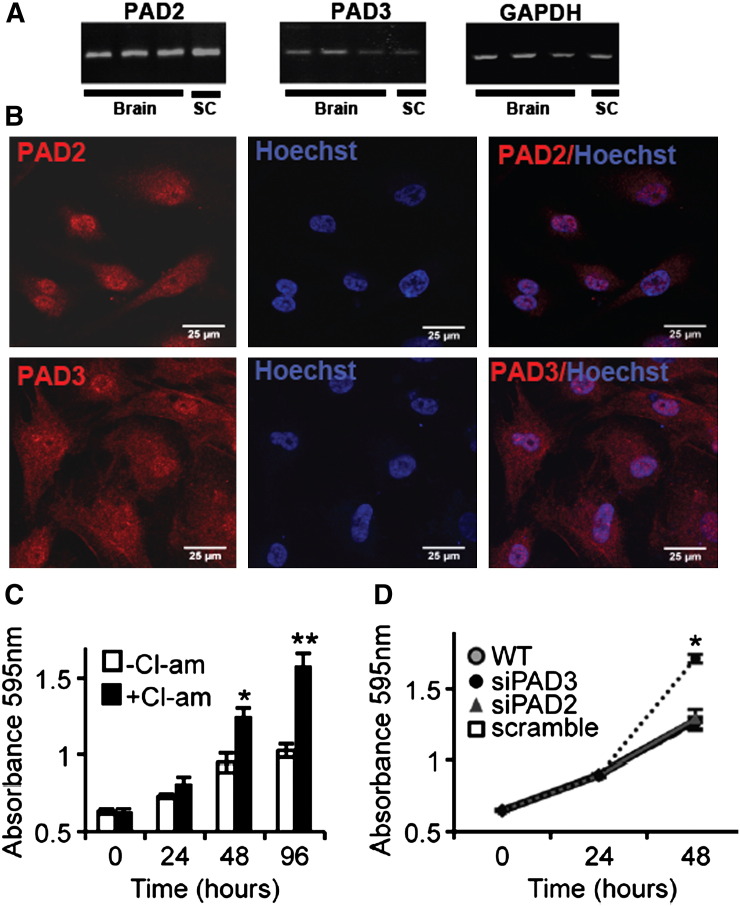
PADs are expressed in human neural stem cells (hNSCs) and PAD3 inhibition increases hNSC proliferation. A) PAD2 and PAD3 transcript detected by RT-qPCR in hNSC derived from embryonic brain and spinal cord (SC). B) Detection of PAD2 and PAD3 by immunocytochemistry (red) in hNSCs: both proteins are detected in cytoplasm and nucleus (counterstained with Hoechst dye). Scale bars: 25 μm. All pictures are at the same magnification. C) Analysis of cell growth determined by the methylene blue assay after treatment with 100 μM Cl-amidine for 24, 48 or 96 h. Cl-amidine significantly increases hNSC proliferation as compared to controls at 48 and 96 h. * = p < 0.05, ** = p < 0.01 by ANOVA and Student's *t*-test. D) Analysis of cell growth determined by the methylene blue assay after transfection with siRNA against PAD2 (siPAD2) and PAD3 (siPAD3) or scrambled siRNA. A significant increase in cell growth as compared to controls is observed at 48 h only in cells transfected with siPAD3 (p < 0.05; two-way ANOVA). Error bars indicate SDM; n ≥ 3.

PAD mRNA expression was assessed in the human central nervous system (CNS), brain and spinal cord, at different developmental stages (Fig. 1). Only PAD2 and PAD3 were detected in the developing brain and spinal cord at all developmental stages studied (46, 63 and 70 days of gestation), whereas the developing human liver, which was used as a control, also expressed PAD1 and PAD4 (Supplementary Fig. S1A). An increase in the PAD2 and a decrease in the PAD3 transcript are observed with development, but both are detected in the brains and spinal cords at all stages of gestation studied. Analysis of PAD2 and PAD3 expression by in situ hybridization at 46 days of gestation is consistent with this finding and shows that PAD3 expression in the developing human spinal cord is particularly high in the germinal zone (Fig. 2B). Antibodies to PAD2 and PAD3, that reacted with proteins of the expected molecular size in Western blots (Supplementary Fig. S1B) were used to assess PAD2 and PAD3 protein expression. Both PAD2 and PAD3 proteins could be detected by Western blot in the developing human brains and spinal cords at the stages tested, but their levels of expression were more difficult to quantify given the limited amount of human material available (Fig. 1C).

PAD mRNA expression was then assessed in 4 hNSCs lines we generated from embryonic human brain and spinal cord (Fig. 2A). All hNSC lines expressed PAD2 and PAD3, but were PAD1 and PAD4-negative (Supplementary Fig. S1A). Immunocytochemistry shows the presence of PAD2 and PAD3 proteins in both the nucleus and cytoplasm of hNSCs (Fig. 2B). Although PAD2 is known to be present in the nucleus [46], this is the first time that human PAD3 has been shown to localize also to the nucleus. Nuclear localization of PAD2 and PAD3 in hNSCs, together with evidence of Cit-H3 in these cells, and previously reported detection of the chick PAD3 in nuclear fractions by Western blot, strengthen mounting evidence that translocation to the nucleus of these PADs does not require a classical nuclear localization signal, that is found only in PAD4 [18,47,48].

### 3.2 PAD inhibition in normal hNSC increases cell growth

We investigated the effect of reducing PAD basal activity on hNSC behavior by monitoring cell growth at different times following treatment with the PAD inhibitor, Cl-amidine. A significant increase in cell growth was observed in Cl-amidine treated hNSCs at 48 and 96 h, with 100 μM being the most effective concentration, suggesting a role for PAD in the modulation of cell growth (Fig. 2C, Supplementary Fig. S2). Increased hNSC growth could also be induced by PAD3 siRNA, but neither by scrambled nor PAD2 siRNA (Fig. 2D). These results suggest a role for PAD3 in the modulation of cell growth.

### 3.3 Cell death and PAD3 expression in hNSCs are induced by increasing intracellular Ca^2 +^

We then wished to establish whether increasing cytoplasmic Ca^2 +^ in hNSC with thapsigargin induced cell death in hNSCs, and mimicked the increase in PAD expression and activity observed in response to injury in the chick spinal cord.

**Fig. 3:**
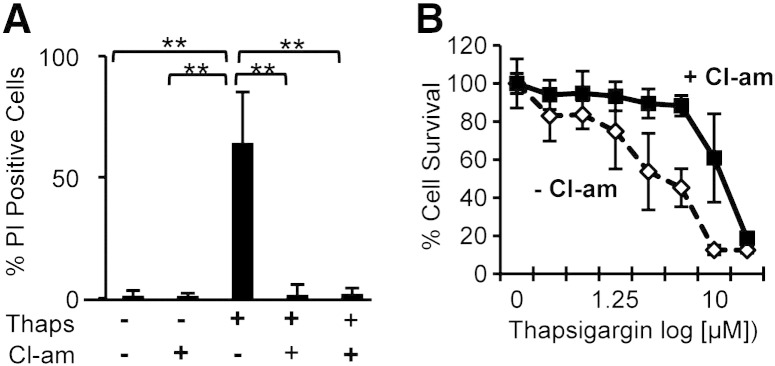
Cl-amidine treatment reduces dose-dependent cell death induced by thapsigargin in hNSCs. A) Quantification of cell death induced by 5 μM thapsigargin (Thaps) detected by propidium iodide (PI) staining. The number of PI-positive cells increases upon thapsigargin treatment and is reduced both by pre-treatment or post-treatment with 100 μM Cl-amidine (Cl-am); n = 8; error bars = s.d.; ** = p < 0.01 by ANOVA and Student's *t*-test. B) Cell survival determined by methylene blue assay after 24 hour treatment with different concentrations of thapsigargin either alone or following 100 μM Cl-amidine treatment (n ≥ 3; error bars = SDM). Cl-amidine treatment significantly increases cell survival (p < 0.05; ANOVA).

The ability of different concentrations of thapsigargin to induce hNSC death at 24 h was assessed by propidium iodide (Fig. 3A and Supplementary Fig. S3) and methylene blue assay (Fig. 3B). Reduced hNSC survival was observed with 5 μM (LD50) thapsigargin and survival was further decreased by increasing concentrations of the compound (Fig. 3B). Thapsigargin-induced cell death was significantly reduced by the PAD inhibitor, Cl-amidine. PAD inhibition also greatly reduced cytoskeleton disassembly induced by thapsigargin, as assessed by staining for actin and vimentin (Supplementary Fig. S4). Altogether this suggests that PAD activation plays an important role in cell homeostasis.

**Fig. 4:**
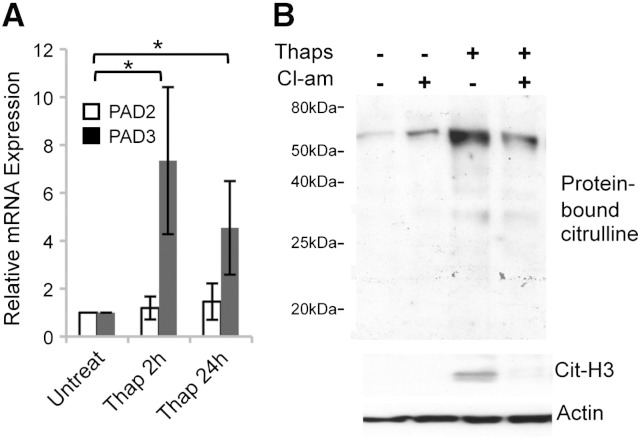
Effect of thapsigargin on PAD expression and citrullination in hNSCs. A) RT-qPCR analysis of PAD2 and PAD3 transcripts after thapsigargin treatment: note up-regulation of PAD3, but not PAD2 transcript, in treated cells; * = p < 0.05 by ANOVA and Student's *t*-test (n ≥ 3; error bars indicate SDM). B) Western blot analysis of citrullinated proteins detected by F95 monoclonal antibody and of citrullinated histone H3 (Cit-H3) following treatment with either Cl-amidine (100 μM) or thapsigargin (5 μM) alone, or both compounds for 24 h. Cl-amidine was added to the culture medium 15 min before thapsigargin treatment. Actin was used as a loading control. Note that PAD activation by thapsigargin results in protein citrullination and this is reduced by the PAD inhibitor, Cl-amidine.

Analysis of PAD expression in thapsigargin-treated hNSC showed a significant increase in PAD3 transcript levels, whereas no change in PAD2 expression was detected (Fig. 4A). Up-regulation of the PAD3 transcript was paralleled by increased protein citrullination, as shown by Western blot using an antibody to protein-bound citrulline. Consistent with PAD nuclear localization we also observed an increase in citrullinated histone 3 (Cit-H3). Thapsigargin-induced protein citrullination was reduced by treatment with Cl-amidine (Fig. 4B). Altogether these data suggest that increased PAD activity in thapsigargin-treated hNSCs is due to PAD3 rather than PAD2.

### 3.4 Cell death is increased by overexpressing PAD3 and reduced by PAD3 inhibition

**Fig. 5:**
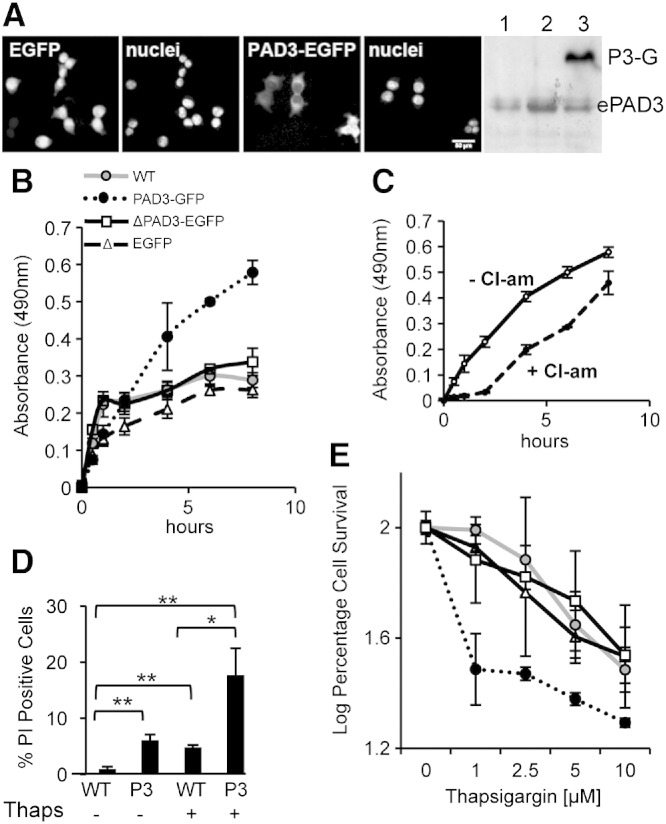
PAD activity is increased in HEK293T cells expressing PAD3-EGFP A) Live images of cells transfected either with EGFP alone or PAD3-EGFP 40 h after transfection with corresponding nuclear staining and detection of endogenous PAD3 (ePAD3) and PAD3-EGFP (P3-G) by Western blot in untransfected cells (1), cell transfected with the EGFP plasmid (2) and cells transfected with PAD3-EGFP. All panels are at the same magnification. Scale bar is 50 μm. B) PAD activity in HEK293T whole cell lysate assessed by the BAEE assay 24 h after transfection. A significant increase (p < 0.05; two-way ANOVA) in PAD activity is observed in HEK293T cell expressing PAD3-EGFP, but not in HEK293T cells expressing the mutated PAD3-EGFP lacking enzymatic activity (ΔPAD3-EGFP) where activity is as in untransfected (WT) cells. C) Cl-amidine (10 μM) significantly (p < 0.05; two-way ANOVA) reduces PAD activity in PAD3-EGFP HEK293T cell lysate in the BAEE assay. D) Cell death determined by propidium iodide (PI) staining 40 h after transfection with PAD3-EGFP (P3); * = p < 0.05, ** = p < 0.01 by Anova and Student's *t*-test. E) Cell survival determined by methylene blue assay after 24 hour treatment with thapsigargin. Significantly (p < 0.05; two-way ANOVA) higher cell death is observed in PAD3-EGFP cells than in HEK293T cells transfected with the EGFP, ΔPAD3-EGFP or WT (labels are as in (B)). Error bars indicate SDM; n ≥ 3.

To further investigate the relative contribution of the PAD isozymes to thapsigargin-induced cell death, we constructed a PAD3-EGFP plasmid and a truncated PAD3-EGFP (ΔPAD3-EGFP) lacking the C-terminal active site of PAD3 (Fig. 5 and Supplementary Fig. S5). We initially tested these construct in HEK293T cells. These cells express PAD3 though at lower levels than hNSCs (Supplementary Fig. S1C). Expression of EGFP control vector and PAD3-EGFP in HEK293T cells was detected as early as 6 h after transfection. It increased over 48 h and PAD3-EGFP was clearly detected by the PAD3 antibody by Western blot, consistent with EGFP detection in live cells (Fig. 5A). Afterward the number of PAD3-EGFP-positive cells, unlike ΔPAD3-EGFP-positive cells, decreased quite rapidly. To ensure that the protein produced by the PAD3-EGFP plasmid was functional, we assessed PAD activity by the BAEE activity assay in protein extracts. PAD activity was much higher in PAD3-EGFP than in ΔPAD3-EGFP and EGFP transfected HEK293T cells, consistent with an active PAD3, and this activity was reduced by Cl-amidine (Fig. 5B, C).

We then assessed the effect of PAD3 over-expression in control and thapsigargin-treated cells. Cell death, as detected by propidium iodide (PI), was higher in HEK293T cells transfected with PAD3-EGFP than in untransfected controls. Upon thapsigargin treatment a significantly higher number of PI-positive cells was observed in PAD3-EGFP as compared to thapsigargin-treated untransfected controls (Fig. 5D). At all the concentrations of thapsigargin tested, cell survival was significantly lower in PAD3-EGFP cells than in cells carrying ΔPAD3-EGFP or EGFP alone (Fig. 5E).

**Fig. 6:**
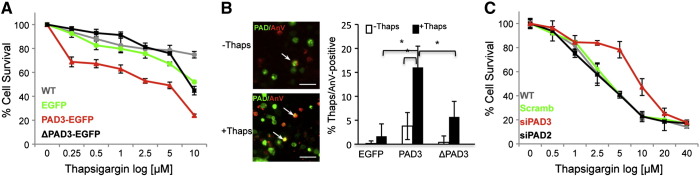
PAD3 is the main PAD involved in hNSC cell death. Death/survival of hNSCs treated for 24 h with thapsigargin was determined by the methylene blue assay and staining for the apoptosis marker annexin V. A) Cell survival is significantly (p < 0.05; two-way ANOVA) reduced in hNSCs carrying PAD3-EGFP as compared to cells transfected with PAD3 lacking the active site (ΔPAD3-EGFP), EGFP alone, or no transfection (WT). B) Example of cells expressing PAD3-EGFP (PAD, green) and Annexin-V (AnV, red) counted for quantification (arrows); note the significantly higher percentage of PAD3-EGFP/Annexin-V-positive cells; * = p < 0.05 by ANOVA and Student's *t*-test. C) Cell survival is significantly (p < 0.05; two-way ANOVA) increased in hNSC transfected with PAD3 but not PAD2 siRNA. Scale bars are 50 μmin B. Error bars indicate SDM; n ≥ 3.

The effect of PAD over-expression in hNSCs was then assessed in hNSC. As for HEK293T cells, PAD3-EGFP reduced survival of thapsigargin-treated cells as compared to cells transfected ΔPAD3-EGFP, EGFP alone, or untransfected (Fig. 6A). Consistent with this observation, a significantly higher percentage of PAD3-EGFP-positve cells, versus cells carrying ΔPAD3-EGFP or an empty EGFP vector control, were annexin-V-positive following thapsigargin treatment (Fig. 6B). To further confirm that PAD3 rather than PAD2 is the main mediator of thapsigargin-induced death, we assessed the effect of PAD3 and PAD2 siRNAs (250 nM) on thapsigargin-induced cell death. Both siRNAs specifically reduced the levels of their respective transcripts by at least 50% as compared to scrambled siRNA or controls (Supplementary Fig. S6); PAD2 siRNA did not show any effect on PAD3 transcript levels, but some effect of PAD3 siRNA on PAD2 levels was observed, though much smaller than that on PAD3. Treatment with PAD3 siRNA rescued thapsigargin-induced cell death, but no such effect was observed with the highly selective PAD2 siRNA, (Fig. 6C). Together these results indicate that PAD3 is the main PAD modulating this response.

### 3.5 PAD3 is involved in AIF translocation and cytoskeleton disassembly

**Fig. 7:**
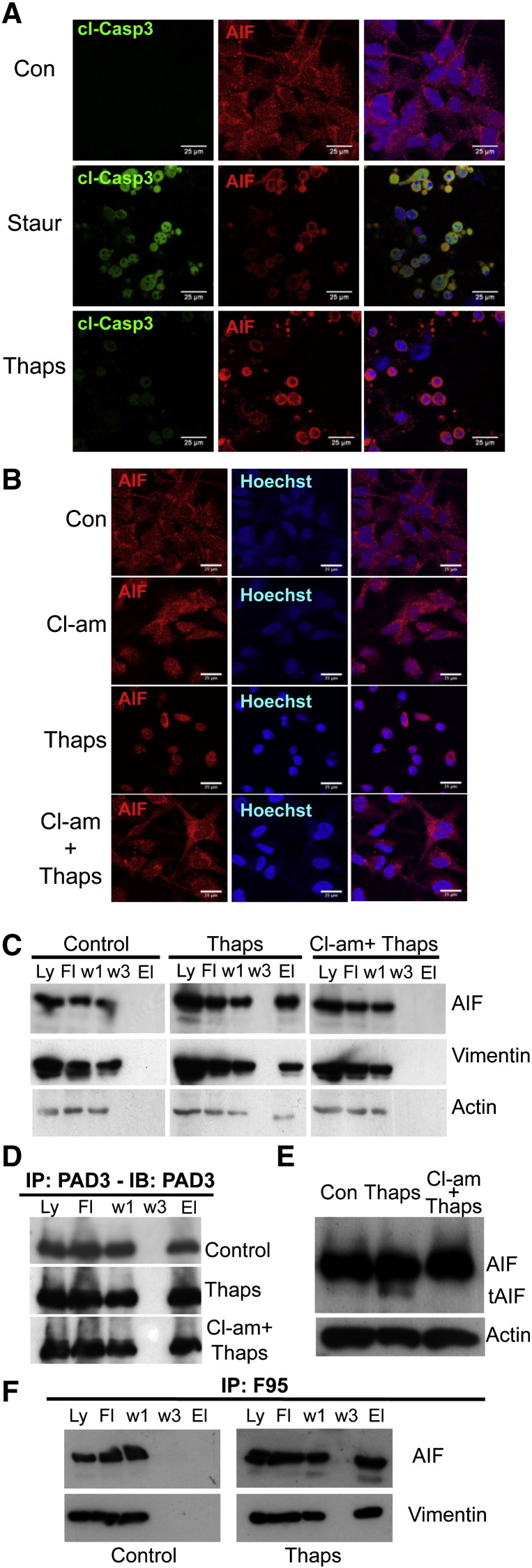
PAD3 expression in hNSCs modulates thapsigargin-induced cell death via AIF translocation. A) Effect of staurosporine (Staur) and thapsigargin (Thaps) treatments for 4 h on caspase 3 activation (cl-Casp3) and AIF translocation to the nucleus assessed by immunocytochemistry and confocal microscopy. Thapsigargin unlike staurosporine does not activate caspase 3, but both induce AIF translocation. B) Confocal images showing translocation of AIF to the nucleus in thapsigargin-treated cells. PAD inhibition by Cl-amidine (Cl-am) blocks AIF translocation. Scale bars in A and B are 25 μm. C) Immunoprecipitation of hNSC protein extract with the PAD3 antibody (Ly: full lysate; Ft: flow through; w1: wash 1; w3: wash 3; El: eluate). Note that AIF, vimentin (Vim) and actin are co-immunopreciptated only in thapsigargin-treated cells. D) PAD3 is immunoprecipitated under all conditions tested; Con: control. E) Western blot of protein extracted from control, thapsigargin- or Cl-amidine and thapsigargin-treated hNSC. The cleaved form of AIF (tAIF) is detected in thapsigargin-treated extracts but not in extract from cells pre-treated with Cl-amidine. F) Immunoprecipitation of control and thapsigargin-treated hNSC protein extracts with the anti-citrullinated protein antibofdy, F95: AIF and vimentin are co-immunoprecipitated only in thapsigargin-treated cells.

In order to identify possible mechanisms of PAD3-induced cell death, we stained thapsigargin-treated cells for cleaved caspase 3 and AIF. This treatment did not induce caspase 3 activation, whereas staurosporine treatment did. In contrast, both treatments induced AIF translocation from the cytoplasm to the nucleus, that was detectable by 2h, with AIF being largely nuclear at 8h (Fig. 7A, Supplementary Fig. 7). Therefore we tested the hypothesis that PAD3-induced cell death is mediated through the AIF pathway. As shown in Fig. 7B, translocation of AIF from the mitochondria to the nucleus was inhibited in thapsigargin-treated hNSCs by the PAD inhibitor, Cl-amidine.

We then wished to gain further insight into the mechanisms by which PAD may regulate cell death. We therefore assessed whether AIF was associated with PAD3. Immunoprecipitation with the PAD3 antibody showed that AIF co-precipitated with PAD3 only in hNSCs treated with thapsigargin, though PAD3 was immunoprecipitated under all conditions tested (Fig. 7C, D). In addition, association of PAD3 with AIF was largely abolished by Cl-amidine pre-treatment, and so was a lower size AIF band observed in thapsigargin-treated lysates. We further assessed the effect of thapsigargin on AIF cleavage by Western blotting (Fig. 7E). The cleaved form of AIF was indeed present in thapsigargin treated-cells, but was not detected when PAD activity was inhibited by Cl-amidine, indicating that PAD activation is required for the release of AIF from the mitochondria. In order to confirm that association of PAD3 with AIF resulted in citrullination of this protein, we investigated whether AIF co-immunoprecipated with the F95 antibody to citrullinated proteins, and this was found to be the case (Fig. 7F). Vimentin, a well-known target of PADs, was also immunoprecipated by F95 (Fig. 7F).

**Fig. 8:**
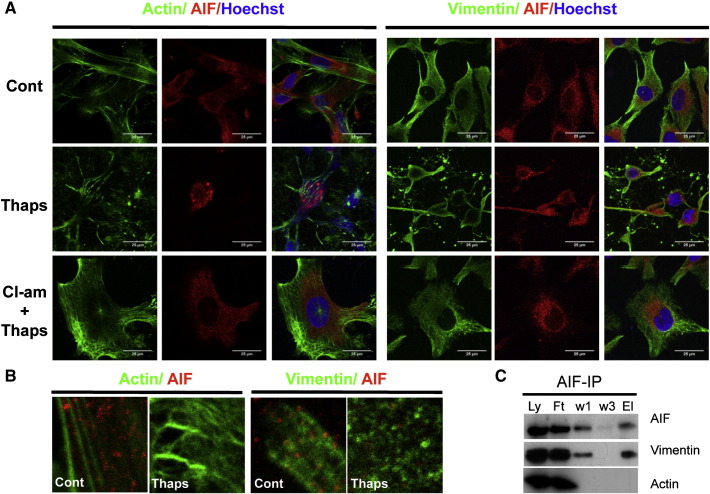
Thapsigargin induces cytoskeletal disorganization in hNSCs that is reduced by PAD inhibition and affects AIF–vimentin association. Confocal images of hNSC stained for actin (detected by phalloidin, green) and vimentin (green) and AIF (red) 3 h after treatment; nuclei are counterstained with Hoechst dye (blue). Double-labeling for actin or vimentin and AIF. Scale bars = 25 μm; all images are at the same magnification. B) High magnification images showing possible association of vimentin and AIF and disorganization of the cytoskeleton upon thapsigargin treatment. C) Immunoprecipitation of hNSC protein extract with the AIF antibody (Ly: full lysate; Ft: flow through; w1: wash 1; w3: wash 3; El: eluate). Vimentin but not actin is co-immunoprecipitated by AIF.

As cytoskeletal protein integrity was affected by thapsigargin (Supplementary Fig. S4), we also assessed whether vimentin and actin, like AIF, were immunoprecipitated by PAD3 only in thapsigargin-treated cells. This was indeed the case, and this association was inhibited by Cl-amidine pre-treatment, that partly restored cytoskeletal organization as well as AIF cytoplasmic localization in the thapsigargin-treated cells (Supplementary Fig. S4, Fig. 7A, C). In these experiments, double immunostaining for AIF and either vimentin or actin in hNSCs suggested a possible co-localization of AIF and vimentin particularly in control cells (Fig 8A–B). We therefore assessed whether there was any direct interaction of AIF with the cytoskeleton by AIF immunoprecipitation. Vimentin, but not actin, was found to be associated with AIF (Fig 8C); this might reflect a role of this intermediate filament in stabilizing AIF in the mitochondria.

## 4 Discussion

Together, the findings reported here suggest that fine tuning of PAD activity is important for hNSC homeostasis, and that modulation of PAD3 levels/activity plays a role in balancing their growth and death. This is consistent with PAD3 association with AIF in the presence of calcium. It has been proposed that identification and targeting of upstream regulators of AIF release would provide a valuable therapeutic approach. Our study suggests that PAD3 is such a regulator.

### 4.1 PAD2 and PAD3 are developmentally regulated in the human CNS

We showed that at embryonic stages and early fetal stages of human CNS development only PAD2 and PAD3 transcripts are detected. This is consistent with PAD4 localization to the myelin sheath in the human CNS, a structure that has not yet formed at the developmental stages that we were able to examine [12]. The overall levels of PAD2 and PAD3 transcript change in opposite fashion with development, with the former increasing and the latter decreasing. This parallel previous observation in the chick embryo where PAD3 in the germinal zone decreases with development and is found in a subset of neurones at later developmental stages.

Expression of PAD3 at early stages of development in the germinal region may seem counterintuitive given the evidence of PAD3 involvement in the apoptotic pathway. As the human embryo is not a very tractable model for functional studies, at this stage we can only speculate on the possible functions of PADs in the developing human CNS. During development, dynamic spontaneous Ca^2 +^ transients in the rat ventricular region have been proposed to play a role in neural progenitor proliferation, apical nuclear migration and early differentiation [32,49]. Brief PAD activation in response to Ca^2 +^ oscillations may therefore be required to modify target proteins involved in some of these processes, but not sufficient for extensive citrullination leading to cell death. This is consistent with the presence of low levels of citrullinated proteins in untreated chick embryos and with evidence that an increase in intracellular Ca^2 +^ lasting at least 10 min is required for AIF translocation leading to cell death [29]. As we have found that PAD inhibition increases hNSC growth, one could hypothesize that cyclical PAD activation in response to physiological Ca^2 +^ transients may contribute to the regulation of neural progenitor proliferation/differentiation program(s).

### 4.2 PAD3 is the main PAD modulating cell death in hNSCs

Both PAD2 and PAD3 are expressed in hNSCs. However, they are differently regulated at the transcriptional level: whereas the PAD3 transcript is up-regulated by increased intracellular calcium, PAD2 is not affected. This difference in human PAD2 and PAD3 regulation by Ca^2 +^ at the transcriptional level in the human cells parallels that observed in the chick following spinal cord injury, where only the PAD3 isozyme was found to be up-regulated in a microarray screen aimed at identifying gene expression changes in response to injury [6]. Ca^2 +^-induced increase in PAD3 transcript levels in neural cells is also consistent with regulation of epidermal PAD3 by Ca^2 +^ in keratinocytes, where extensive analysis of PAD3 transcriptional regulation has been carried out [50–53].

Inhibition of PAD3 activity reduces thapsigargin-induced cell death in human cells and this is consistent with the in vivo injury response reported in the chick [6]. We have several lines of evidence supporting the hypothesis that PAD3 and not PAD2, which is the other PAD present in hNSCs, mediates Ca^2 +^-induced cell death. Increased cell death is observed in cells over-expressing PAD3, and PAD3 siRNA can significantly reduce thapsigargin-induced cell death. In contrast, in these experiments PAD2 knockdown does not increase cell survival in treated cells, clearly suggesting different functions for these two PADs in hNSCs.

### 4.3 PAD3 is a key upstream regulator of AIF-dependent/caspase 3-independent cell death

Thapsigargin is a Ca^2 +^-ATPase inhibitor that increases cytoplasmic Ca^2 +^ by reducing its up-take into the endoplasmic reticulum stores. As shown here, thapsigargin induces cell death in hNSCs in a caspase 3-independent manner. In contrast, thapsigargin treatment promotes translocation of AIF (apoptosis inducing factor) to the nucleus, a pathway known to be activated by increased intracellular Ca^2 +^. AIF is a mitochondrial intermembrane flavoprotein that plays a vital role in mitochondrial function in addition to its role in cell death execution, where, upon translocation from the mitochondria to the nucleus and binding to DNA, induces chromatin condensation and DNA degradation [54,55]. AIF-mediated cell death depends on the cell type and nature of the apoptotic insult, which may determine the mechanisms of AIF release from the mitochondria [56–59]. Significantly, AIF has been reported to play a role in neural tissue damage following hypoxia ischemia in neonates, and there is some evidence of neural damage reduction in the neonatal hypoxic–ischemic model upon PAD inhibition [7,60].

It has been proposed that the calcium-dependent activation of the cysteine protease calpain is required to cleave AIF, that is then released from the mitochondrial membrane [29,61]. We have shown that in hNSCs, PAD activation is required for AIF cleavage consistent with AIF co-precipitating with PAD3 in thapsigargin-treated cells and its inhibition by Cl-amidine. This positions PAD signaling upstream of AIF release. It is conceivable that AIF citrullination is required to expose AIF to calpain as suggested for filaggrin and NSE and this will require extensive investigation [3,62]. Furthermore, vimentin, a well-known PAD target that we have shown here to become associated with PAD3 when cytoplasmic Ca^2 +^ is increased, has also been reported to be cleaved by calpain and to play a role in cell death [27].

It is tempting to speculate that citrullination of vimentin may play a role in AIF translocation. Several cytoskeletal proteins are known to be PAD targets, and in the oocyte PAD6 is required for lattice formation and regulation of ribosomal component movements [19,63]. Vimentin is involved in several cellular processes, including mitochondria motility and apoptosis [2,27,64–67]. The role of vimentin association with AIF has yet to be elucidated, but it is conceivable that vimentin destabilization upon citrullination could affect the mitochondria facilitating AIF release from these organelles and its subsequent translocation to the nucleus. A possible role for PAD3 in regulating translocation of a mitochondrial-associated protein to the nucleus is also interesting in the context of recent work showing a role for citrullination in protein trafficking in the opposite direction, where REF (RNA binding export factor) citrullination appears to be required for ATP5 mRNA transport to the mitochondrial surface [1].

Extensive investigation will be required to assess the role of the cytoskeleton in AIF translocation and the precise role of PAD in this process and in AIF cleavage. Nonetheless, altogether our results clearly position PAD3 as an early player in the cascade of events leading to Ca^2 +^ dependent/caspase 3-independent cell death in hNSCs.

Finally, it has been suggested that AIF-mediated neural cell death is of particular importance in neural tissue and in some tumors [56,58,68]. Information on PAD3 localization in adult human CNS is lacking, but PAD3 mRNA has been detected in the human cerebellum [21]. Therefore it will be important to establish whether PAD3 expression determines AIF-mediated cell death restriction to specific neural cell subpopulations in pathologies involving increase in intracellular Ca^2 +^. Future studies on PAD3 expression in normal and pathological human specimen will be crucial to clarifying the role of this isozyme in neural pathologies.

## 5 Conclusions

We have shown that human PAD3 (PADI3) is a key player in hNSC homeostasis, and particularly in caspase 3 independent cell death induced by increased intracellular calcium in hNSCs. This to our knowledge is the first study that demonstrates that PAD3 is an upstream regulator of Ca^2 +^-induced cell death in human neural cells and sheds some light on the mechanisms involved. Furthermore, it highlights differences in the role of PAD2 and PAD3 in hNSCs. Together with our previous work in animal injury models, our findings in a human model identify PAD3 pathway targeting as a novel approach to reducing neural tissue loss in human pathologies where increased intracellular Ca^2 +^ greatly contributes to cell damage, such as traumatic and hypoxic/ischemic insults to the CNS.

The following are the supplementary data related to this article.

Table 1 Primer sequences used for RT-PCR and qRT-PCR (*).

Supplementary figures.

Supplementary data to this article can be found online at http://dx.doi.org/10.1016/j.bbamcr.2014.02.018.
