# Supplementary material for: Modulation of calcium-induced cell death in human neural stem cells by the novel peptidylarginine deiminase–AIF pathway
Source: Biochim Biophys Acta. 2014 Jun;1843(6):1162–71. doi: 10.1016/j.bbamcr.2014.02.018 (PMC3996523; doi:10.1016/j.bbamcr.2014.02.018)
Supplement: Table 1 — Primer sequences used for RT-PCR and qRT-PCR (*). [file mmc1.doc]

**Supplementary Information**

**Table 1.** Primer sequences used for RT-PCR and qRT-PCR(*)

| **Gene** | **Forward Primer** | **Reverse Primer** | **T** | **bp** | **f-exon** |
| --- | --- | --- | --- | --- | --- |
| PAD1 | CGCCATCCTCTCTGCCCTCTTGCTA | GGTTTTTCTGTCCTTGTTTGTCCAC | 58 | 571 | Exon 16 3’UTR |
| PAD2 | ATGCACCTTCATCGACGACATTT | TTTCAGCAGGGACAGAGTCGAG | 54 | 332 | Exon 16 |
| PAD3 | GCAGAGTGTGACATCATTGACATCC | GACCGCACCTTCTCCTCCAG | 68 | 173 | Exon 15-16 |
| PAD4 | TCTTGTGAATATTGTGGCTCCCT | AGAGCAGAACTGAGTGTGCAGTG | 56 | 134 | Exon 16- 3’UTR |
| PAD2* | TGAAGCACTCGGAACACGT | TTGTCACTGCTGGCCTCG | 59 | 150 | Exon2 |
| PAD3* | AATGTTTGAGGTCTATGGGA | CCAAAGTCGCGTCAAAGC | 59 | 109 | Exon 2 |
| GAPDH | CCTTCATTGACCTCAACTACATGGT | CTAAGCAGTTGGTGGTGCAGGA | 56 | 356 | Exon 3-7 |

T : annealing temperature(°C). bp: Product size; F-exon: flanking exon. The same GAPDH primers were used for RT-PCR and qRT-PCR.
