# Supplementary material for: Modulation of calcium-induced cell death in human neural stem cells by the novel peptidylarginine deiminase–AIF pathway
Source: Biochim Biophys Acta. 2014 Jun;1843(6):1162–71. doi: 10.1016/j.bbamcr.2014.02.018 (PMC3996523; doi:10.1016/j.bbamcr.2014.02.018)
Supplement: Supplementary file 2 — Supplementary figures. [file mmc2.pdf]

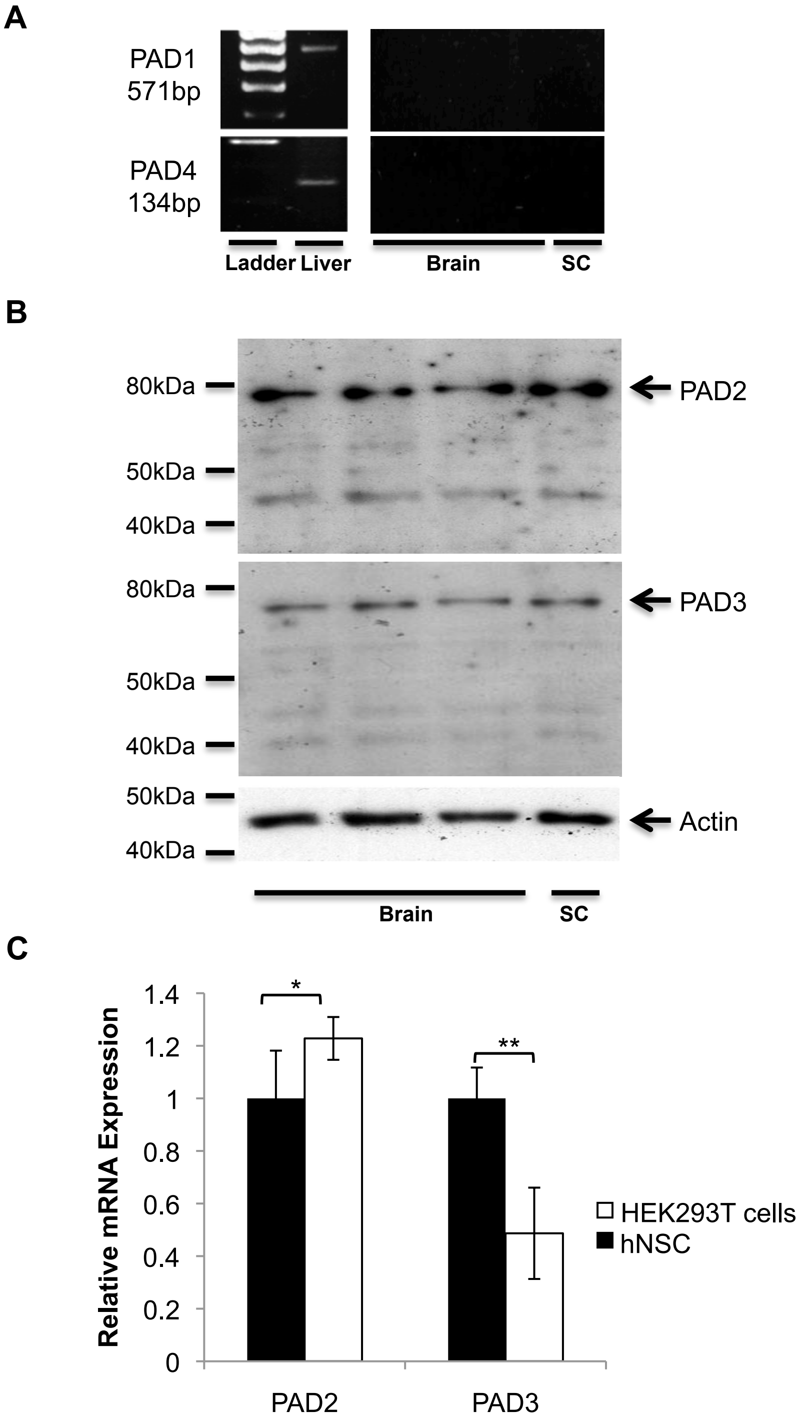

Supplementary Figure S1: Detection of PADs expression in hNSC lines derived from human embryonic brains and spinal cord. A) Detection of PAD1 and PAD4 by RT-PCR in hNSC lines. Human fetal liver is used as a positive control and all hNSC lines are negative for PAD1 and PAD4. **B)** Detection of PAD2 and PAD3 protein by Western blot in hNSC lines. Protein of the expected molecular weight are detected by the antibodies to PAD2 and PAD3. **C)** Quantitative RT-PCR comparing PAD2 and PAD3 expression in HEK293T and hNSC. HEK293T cells express higher level of PAD2, while hNSC express higher level of PAD3; * = $p<0.05$, **= $p<0.01$ by Anova and Student's t-test ($n\geq3$; error bars indicate SDM).

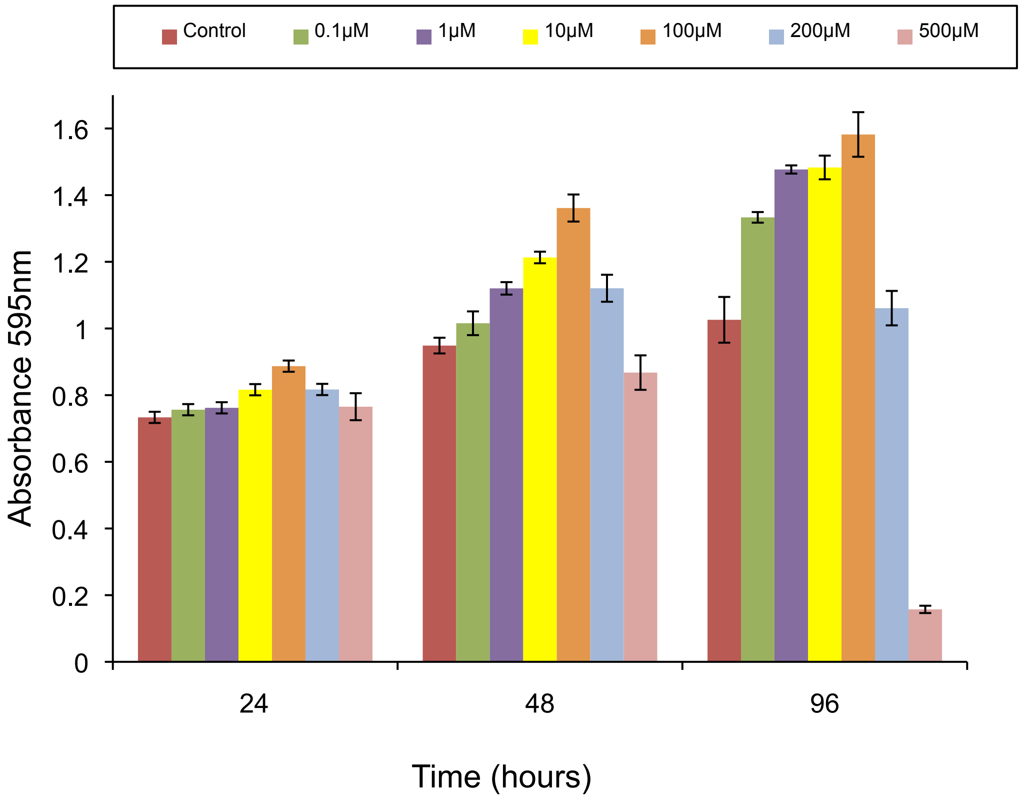

Supplementary Figure S2. Dose-dependent effect of Cl-amidine on hNSC growth. Analysis of cell growth determined by the methylene blue assay after treatment with different concentrations of Cl-amidine for 24, 48 or 96 hours. Cl-amidine significantly increases hNSC proliferation as compared to controls at 48 and 96 hours with maximum effect observed at 100 μM (Anova and Student's t-test; $p>0.05$). At higher concentrations, particularly 500 μM, Cl-amidine significantly affects cell survival as clearly apparent after 96 hours treatment ($p<0.01$). Error bars indicate SDM; n=3.

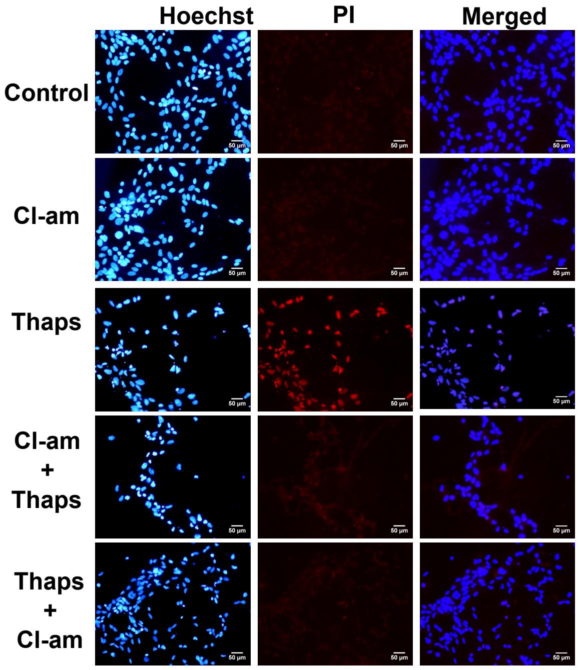

Supplementary Figure S3: Thapsigargin-induced cell death in hNSCs is rescued by the PAD inhibitor Cl-amidine. Cell death is detected by propidium iodide (PI) staining (red) and nuclei by Hoechst dye (blue). Cl-amidine was added either 15 minutes before (Cl-am+Thaps) or after (Thaps+Cl-am) thapsigargin.

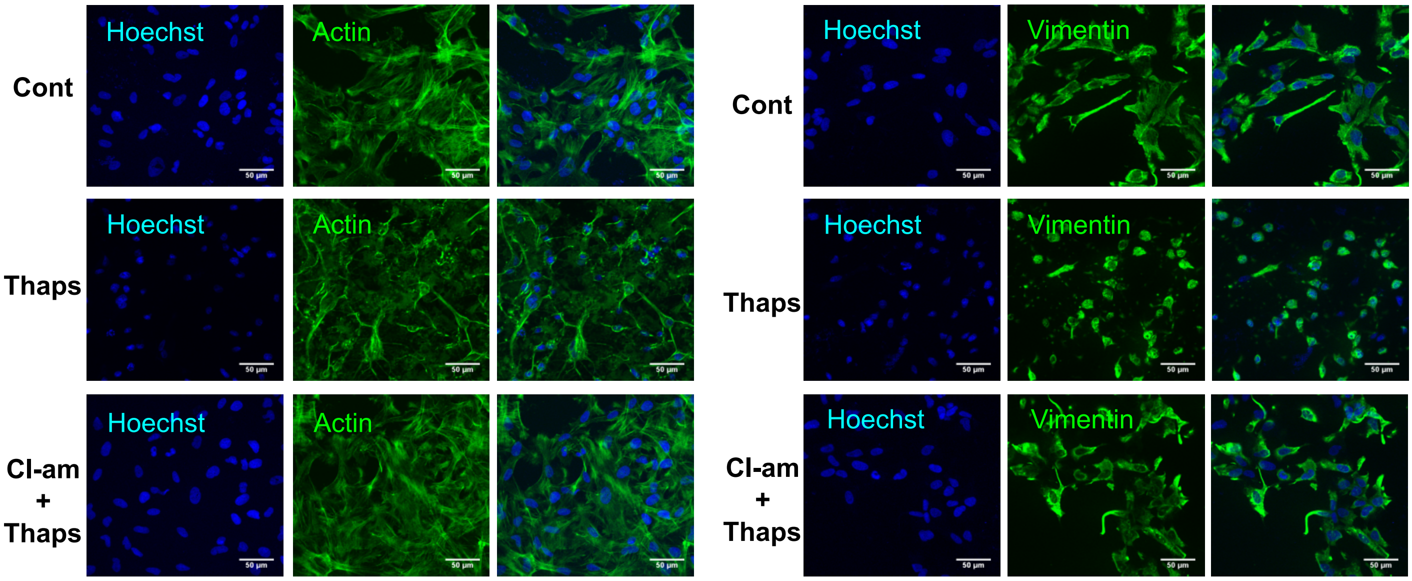

Supplementary Figure S4: Thapsigargin-induced disassembly of actin and vimentin in hNSC is rescued by the PAD inhibitor Cl-amidine. Actin and vimentin detected by phalloidin binding and immunocytochemistry, respectively (green), and nuclei stained with Hoechst dye (blue) three hours after thapsigargin treatment. Cl-amidine was added 15 minutes before thapsigargin (Cl-am + Thaps).

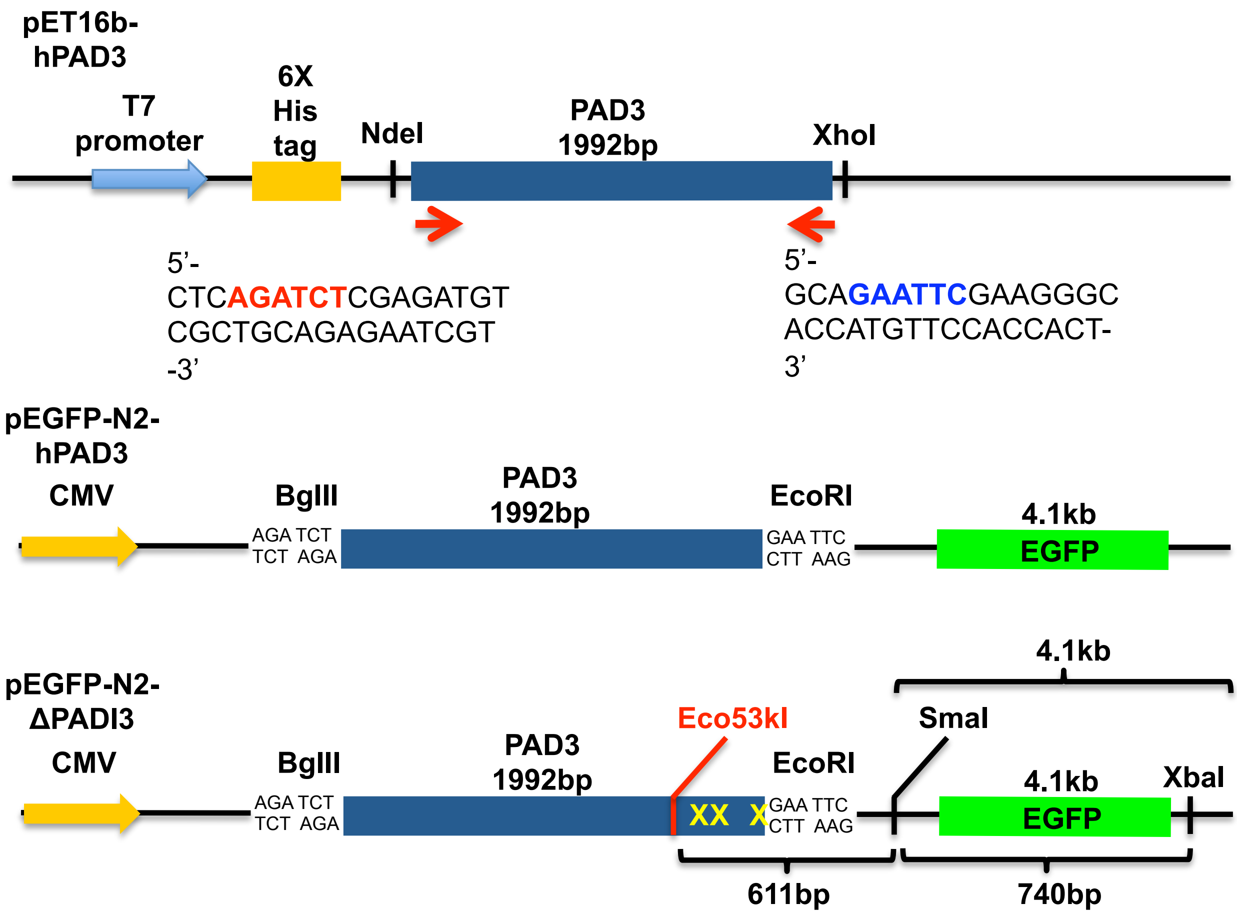

Supplementary Figure S5: Construction of pEGFP-N2-hPAD3 and pEGFP-N2-ΔPADI3. hPAD3 has been amplified from the pET-16b-hPAD3 and cloned into pEGFP-N2 plasmid using the indicated primer sequences. The restriction sites for BglII and EcoRI within the primers are indicated in red and blue, respectively. The hPAD3 PCR product containing flanking BglII and EcoRI restriction sites was ligated into pEGFP-N2 plasmid to produce the pEGFP-N2-hPAD3 construct. pEGFP-N2-hPAD3 was sequentially digested with Eco53kI and SmaI restriction endonucleases to remove the C-terminus region (pEGFP-N2-ΔPADI3) that contains the enzyme active site (yellow X).

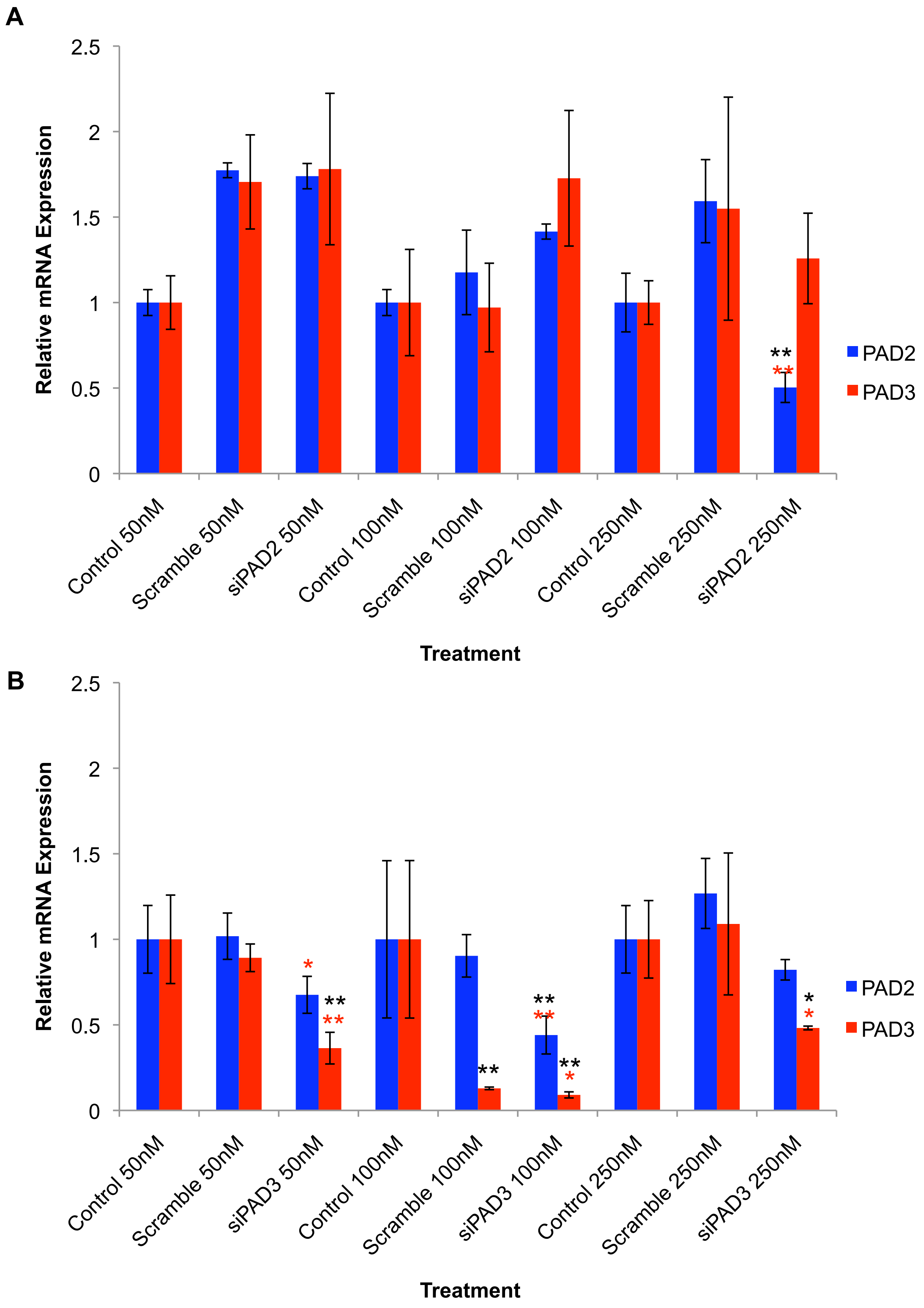

Supplementary Figure S6: Analysis of PAD expression in hNSCs after siRNA treatment at different concentration. A) RT-qPCR analysis of PAD2 and PAD3 transcripts after PAD2 siRNA transfection. **B)** RT-qPCR analysis of PAD2 and PAD3 transcripts after PAD3 siRNA transfection; black asterisks: comparison with controls and red asterisks: comparison with scrambled siRNA by Anova and Student's t-test; * = $p < 0.05$, ** =$p < 0.01$.

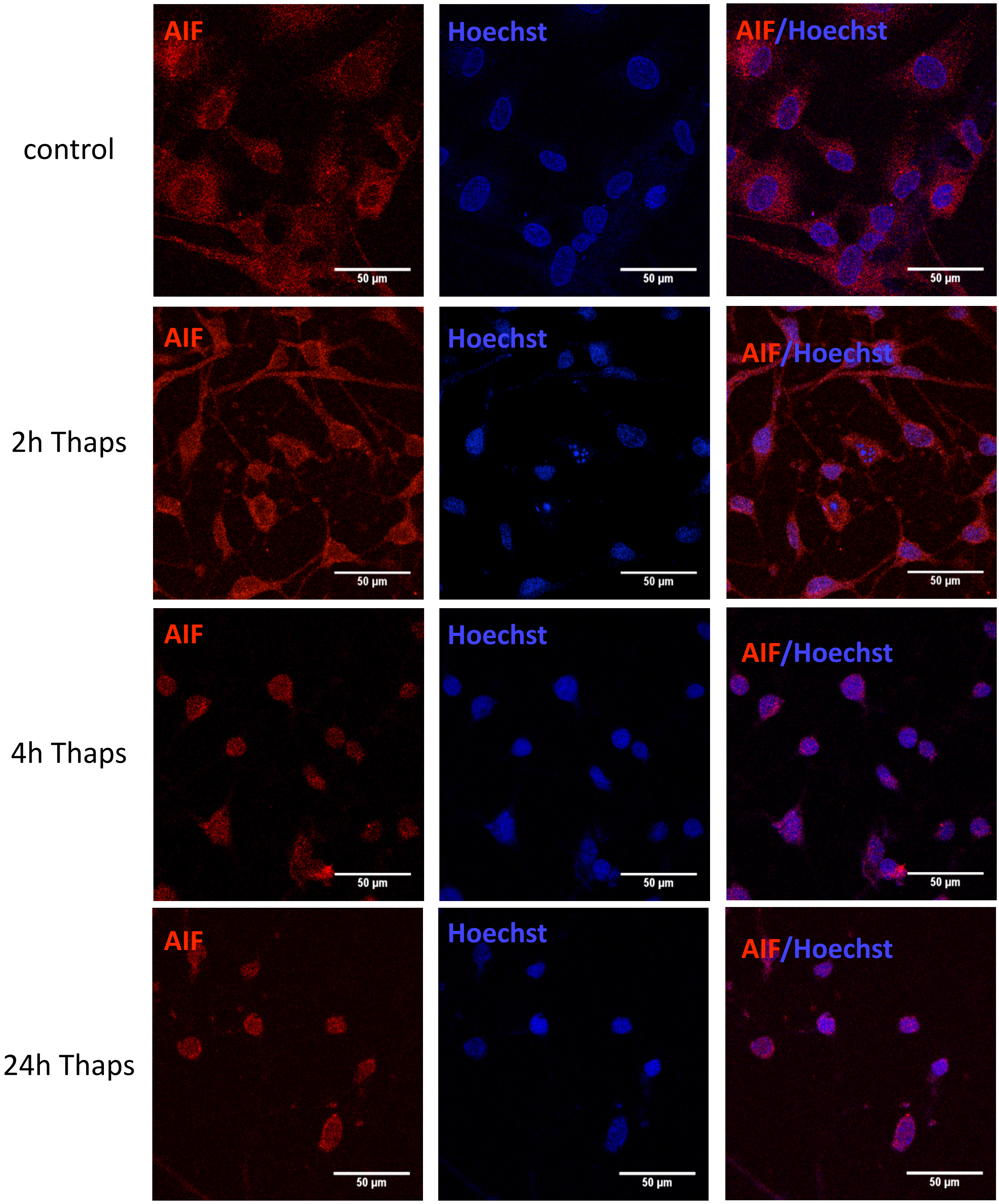

Supplementary Figure S7: Time-course of thapsigargin-induced AIF translocation in hNSCs. hNSCs were stained for AIF 2, 4 and 24 hours after treatment with 5 µM thapsigargin (Thaps). Significant translocation of AIF to the nuclues is observed by 4 hours, and by 24 hours hardly any AIF staining is observed outside the nucleus.
